# A rare case of primary sinonasal meningioma: A case report

**DOI:** 10.1016/j.ijscr.2022.107620

**Published:** 2022-09-09

**Authors:** Meherzi Abir, Lahmar Rihab, Mouna Bellakhdhar, Omri Malika, Hwass Jihen, Kermani Wassim, Mohamed Abdelkefi

**Affiliations:** University of Sousse, Faculty of Medicine of Sousse ENT Department, Farhat Hached Teaching Hospital, Sousse, Tunisia

**Keywords:** Case report, Nasal obstruction, Sinonasal meningioma, Endoscopic approach

## Abstract

**Introduction and importance:**

Extra cranial primary meningioma of sinonasal tract is a rare entity. It is often misdiagnosed as nasal polyp.

**Case presentation:**

Here we report a case of a primary ethmoid sinus meningioma with extension into the nasal cavity in a 41-year-old man. The tumor was completely excised via endoscopic endonasalapproach and the histologic diagnosis of meningioma was established. The patient was regularly follow up for 12 months without recurrence of the tumor.

**Clinical discussion:**

The final diagnosis of primary sinonasal meningioma is based on histopathology and immunohistochemistry analyses results. The importance of complete surgical resection is undoubted and also is a goodindicator prognosis.

**Conclusion:**

The otolaryngologists should be aware of the diagnosis of primary meningioma; despite of its rarity it is considered as a possible cause of nasal obstruction.

## Highlights

• Primary extra cranial meningioma is rare and its Histogenesis is still unknown.
• Clinical presentation of sinonasal ectopic meningiomas is not specific.
• Diagnosis is based on histopathological examination and immunohistochemistry.
• Complete resection is not always possible due to complex anatomy of nasal cavity and paranasal sinuses.

## 1 Introduction

Meningioma is a central nervous tumor that arises from the meninges [1]. Primary extracranial meningioma occurs relatively rarely in less than 1 %; the most common localizations are the skull bones as well as scalp, sinonasal tract, orbit and ear [1], [2], [3]. Meningioma involving the sinonasal tract may mimic nasal polyps. The final diagnosis rests in histological and imunohistochemical examination [2]. The world health organization of the central nervous system tumors has classified these tumors into WHO types I, II, III and 15 subtypes [4].

We report a rare case of primary sinonasal meningioma in an adult man who presented to our hospital with nasal obstruction.

Our work has been reported in line with the SCARE 2020 criteria [5].

## 2 Case report

A 41-year-old man presented to our department with progressive right-sided nasal obstruction, facial pain and intermittent epistaxis for the last 5-months duration. He was not currently taking any medications and had no specific medical history.He was cigarette smoker. There were no history of fever, rhinorrhea or loss of smell. Past and familial history are not significant.

**Fig. 1:**
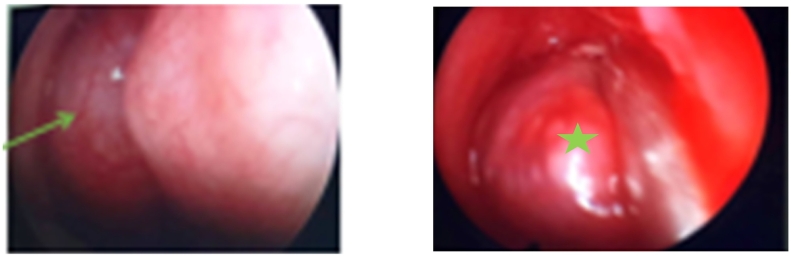
A-B: Endoscopic view showing a well circum scribed mass filling the right cavity nasal.

General and neurologic examinations were unremarkable. Local examination showed no local rupture or uplift of the nasal skin. Anterior rhinoscopy showed awellcircumscribedsoft tissue mass filling the right nasal cavity and pushing the septum to the left (Fig. 1A-B).

The examination of oral cavity and oropharynx was found to be normal. He had no palpable cervical lymph nodes. In fact, the clinical presentation was not specific.

Routine tests were normal.

**Fig. 2:**
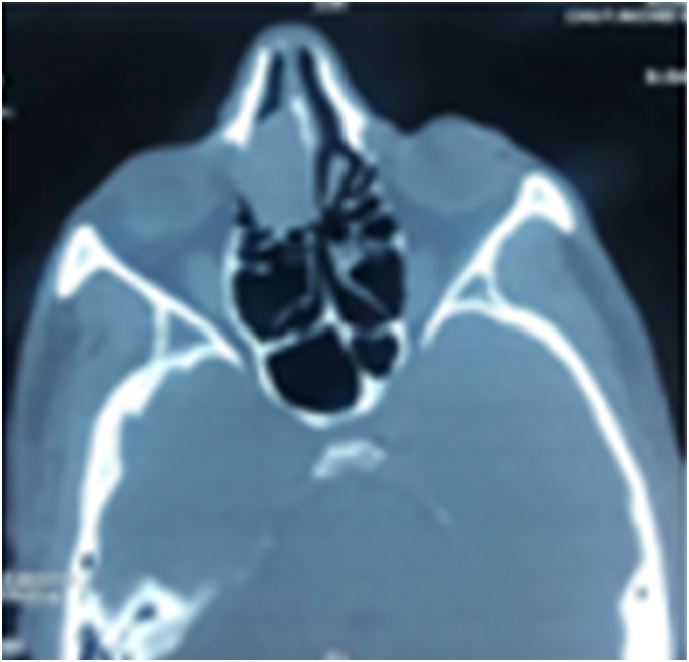
CT-scan axial view showing a homogeneous mass of 27 ∗ 14 ∗ 24 mm involving the right nasal cavity with erosion of adjacent bone.

The CT-scan of the nasal cavity and paranasal sinuses showed homogeneously enhancing mass in the right nasal cavity. It was a 27 ∗ 14 ∗ 24 - mm ovoid mass with extension into the right ethmoid sinus superiorlyandwith erosion of adjacent bone (Fig. 2).

**Fig. 3:**
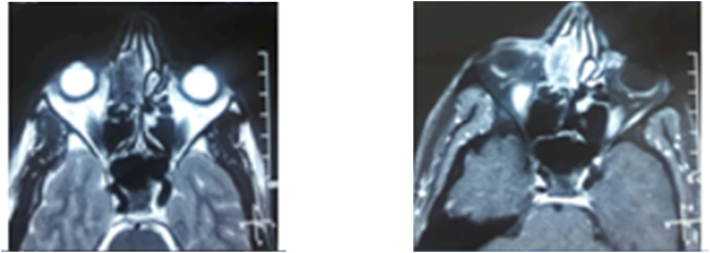
A-B: MIR axial view showing a tissue process of the right nasal cavity with extension into the ipsilateral anterior ethmoid sinus; in heterogeneous hyposignal T2 (A) with homogeneous intense enhancement after injection of Gadolium (B).

Magnetic imaging resonance (MIR) was performed ruling out intracranial connection or intraorbital extension (Fig. 3A-B).

**Fig. 4:**
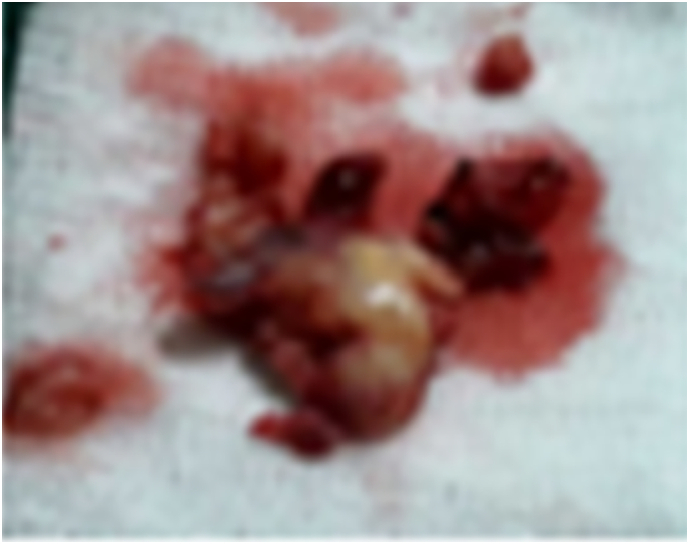
Meningioma after endoscopic excision.

The decision was to proceed by an endoscopic approach. No special preoperative preparation was required. The patient was taken to the operating room. He was operated on by a senior ENT surgeon after giving his informed consent. Peroperative findings revealed a friable and vascular mass occupying the right nasal cavity. The mass was completely resected using endoscopic endonasal surgical approach under general anesthesia (Fig. 4). The postoperative time went without incident. The patient was given antibiotics for 48 h along with analgesics and care for the nasal cavity.

Histologic examination revealed a well circumscribed growth composed of spindly cells with oval bland nuclei and moderate amount of eosinophilic cytoplasm. No nuclear atypia, mitoses, necrosis or invasion of the soft tissue were seen. Tumor cell vimentin, S100 protein (à verifier) and Ki-67 were positively expressed. Negative staining was seen with cytokeratins, CD34, CD31, CD68 and alpha-1 anti-trypsin. Features were suggestive of a WHO grade I nasal meningioma. The post-operative course was simple with no complications or adverse outcomes.

The patient was regularly followed up in our outpatient clinic every three months for a total of three years following the operation. Imaging studies and clinical examinations have not revealed any tumor recurrence.

## 3 Discussion

Meningioma is a nonglialtumor of the central nervous system, representing 24–30 % of all intracranial tumors. It arises from arachnoid ‘cap cells’ or meningocytes that is derived from the neural crest [1], [6]. It is more common in women and it can occur in all ages [4].

Primary extra cranial meningioma is rare, accounting for less than 1 % of cases [2], [4]. Our study reports a case of primary sinonasal meningioma in an old man.

The histogenesis is still unknown. They may originate frommeningothelial cells that are displaced into this region during embryogenesis [7], [8].

The clinical manifestations of sinonasal ectopic meningiomas reported in literature are nonspecific and usually include nasal obstruction, anosmia, facial pain, nasal discharge, epistaxis and facial deformities [1], [4].

Nasal endoscopy usually shows a firm, pink to gray mass filling the nasal cavity. Meningiomas are often well circumscribed, globular or lobulated tumors without infiltration into surrounding tissues [1], [8], [9], [10].

CT-scan or MIR is unable to identify the nature of the nasal cavity tumor, but it is essential in determining the location, size and it gives information of the extent and invasion of the tumor; compression and deformation can be observed in adjacent bones. Calcification is rare, but no specificity is present [2], [4].

Radiological findings in our case were also nonspecific. Therefore, primary sinonasalmeningiomas have been often misdiagnosed as inverted papilloma or nasal polyps [1], [11].

WHO (World Health Organization) has classified meningioma into 3 grades; the typical or benign type (Grade I), the atypical with frequent mitosis (Grade II) and the anaplastic type with invasion (Grade III) [2], [3], [11]. Most of sinonasalmeningiomas are benign; local infiltration as well as distant metastasis may occur in malignant forms [2].

In our case, the slow growth of the tumor as well as lack of perineural and adjacent structures invasion were in favor of the benign nature.

Diagnosis is based on histopathological examination and immunohistochemistry; meningiomas are so immunoreactive to vimentin, EMA (epithelial membrane antigen) and pancytokeratin. Some meningiomas also show positive reactions to S100 protein, CK7, CK20, Ki-67, synaptophysin, CD34, estrogen and progesterone receptor [8], [11], [12], [13].

Surgical resection is the treatment of choice for the sinonasal meningioma, but complete resection is not always possible due to complex anatomy of nasal cavity and paranasal sinuses [1], [4], [13].

Endoscopic approach allows satisfactory view of the operative field and complete tumor resection. It is correlated with better prognosis. It is less invasive to the surrounding healthy tissue than classical approach [2].

Efficacy of adjuvant radiotherapy is not established, it should be considered in case of incomplete tumor resection or malignant transformation [10].

In our case we achieved a complete resection of the tumor and radiotherapy was not needed.

Y. Liu, H et al. studied the relationships between histological grade and recurrence in 170 cases; recurrence was found to be 8 % in benign tumor [1], [6].

K. Sharma et al. achieved a 5-year disease- free survival rate at 72 % [2].

## 4 Conclusion

Primary sinonasalmeningiomas are rare condition. Most of them are benign; only few malignant meningiomas have been reported.

It should be considered as one of differential diagnoses of nasal polyps and inverted papilloma.

The diagnosis requires histopathological and immunohistochemical examination. Endoscopic approach is very safe and effective approach to resect these tumors completely.

(Treatment of these tumors is based on surgical resection using usually an endoscopic approach).

## Sources of funding

We have no sources of funding for our research.

## Ethical approval

This study is exempt from ethnical approval in our institution.

## Consent

We have obtained a written and signed consent to publish this case reports from the patient prior to submission.

## CRediT authorship contribution statement

MEherzi Abir: conception and design of the study.

Omri Malika, Hwass Jihen: acquisition of data.

Meherzi Abir, Lahmar Rihab: article drafting.

Wassim Kermani, Mouna Bellakhdher, Mohamed Abdelkefi: final approval of the version to be submitted.

## Research registration

Not applicable.

## Guarantor

Dr. Meherzi Abir.

## Declaration of competing interest

We declare that we have no conflicts of interest related to this article.
